# Some properties of a T operator with B-M kernel in the complex Clifford analysis

**DOI:** 10.1186/s13660-018-1816-6

**Published:** 2018-08-30

**Authors:** Zunfeng Li, Yuying Qiao, Nanbin Cao

**Affiliations:** 1 College of Science, Hebei University of Science and Technology, Shijiazhuang, P.R. China; 2 College of Mathematics and Information Science, Hebei Normal University, Shijiazhuang, P.R. China; 3 Hebei GEO University, Shijiazhuang, P.R. China

**Keywords:** Complex Clifford analysis, Teodorescu operator, Boundedness, Hölder continuity, *γ*-integrability

## Abstract

Teodorescu operator, or T-operator, plays an important role in Vekua equation systems and the generalized analytic function theory. It is a generalized solution to the nonhomogeneous Dirac equation. The properties of T operators play a key role in the study of boundary value problems and integral representation of the solutions. In this paper, we first define a Teodorescu operator with B-M kernel in the complex Clifford analysis and prove the boundedness of this operator. Then we give an inequality similar to the classical Hile lemma about real vector which plays a key role in the following proof. Finally, we prove the Hölder continuity and *γ*-integrability of this operator.

## Introduction

In some way, there are two branches of Clifford analysis. The first one is the real Clifford analysis introduced by Brack, Delanghe, and Sommen in [1] which studied function theory with values in a real Clifford algebra defined on a nonempty subset of the Euclidean space $R^{n+1} $. Many important theoretic results, such as the Cauchy integral formula, the Cauchy theorem, the Taylor and the Laurent series expansion, the Liouville theorem, and the Morera theorem, have been obtained, and they are the extensions of the well-known classical theorems in one complex variable. Beyond these, a lot of scholars have studied many properties of function theory in the real Clifford analysis. Eriksson and Leutwiler [2–5] introduced the hypermonogenic function and studied some properties of it. Huang [6], Qiao [7–9], Xie [10–12], and Yang [13–15] obtained many results in Clifford analysis.

The second one is the complex Clifford analysis. In the early 1990s, Ryan [16–19] introduced the definition of the complex regular function and obtained the Cauchy integral formula whose method is similar to the classical function with one complex variable. In recent years, Ku, Du [20, 21] obtained some properties of complex regular functions using the isotonic function.

Based on the above theoretical study and practical background, we construct an analogue of Bochner–Martinelli kernel in several complex variables. We first define a Teodorescu operator with B-M kernel in the complex Clifford analysis and prove the boundedness of this operator. Then we give an inequality similar to the classical Hile lemma about real vector which plays a key role in the following proof. Finally, we prove the Hölder continuity and *γ*-integrability of this operator.

## Preliminaries

Let $\mathrm{Cl}_{0,n}(C)$ be a complex Clifford algebra over n+1-dimensional Euclidean space ${\mathbf{C}}^{n+1}$. $\mathrm{Cl}_{0,n}(C)$ has the basis $e_{0}, e_{1}, e_{2}, \ldots, e_{n}; e_{1}e_{2}, e_{1}e_{3}, \ldots, e_{1}e_{n}; e_{2}e_{3}, \ldots, e_{2}e_{n}; \ldots; e_{n-1}e_{n}; \ldots; [4] e_{1}\cdots{e_{n}}$. Hence, an arbitrary element of the basis may be written as $e_{A}=e_{\alpha_{1}}\cdots e_{\alpha_{h}}$, where $A=\{\alpha_{1}, \ldots, \alpha_{h} \}\subseteq\{1, \ldots, n\}$, $1\le\alpha_{1}<\alpha_{2}<\cdots<\alpha_{h}\le n$ and when $A=\emptyset$, $e_{A}=e_{0}=1$. So, the complex Clifford algebra is composed of elements having the type $a=\sum_{A}z_{A}e_{A}$, where $z_{A}$ are complex numbers.

The basis in Clifford algebra satisfies

$${e_{i}^{2}=-1,\quad i=1, 2, \ldots, n}, \qquad e_{i}e_{j}=-e_{j}e_{i},\quad 1\leq i< j\leq n, (i\neq j). $$

Define the norm of Clifford numbers as follows:

$$\biggl\vert \sum_{A}z_{A}e_{A} \biggr\vert =\sqrt{(a, a)}= \biggl(\sum_{A} \vert z_{A} \vert ^{2} \biggr)^{\frac{1}{2}}. $$

Let $\Omega\subset\mathbf{C}^{n+1}$ be an open connected nonempty set. Then the function which is defined on Ω and valued in $\mathrm{Cl}_{0,n}(C)$ can be expressed as $f(z)=\sum_{A}{f_{A}(z)e_{A}}$, where $f_{A}(z)$ are complex-valued functions. Let

$${F^{(r)}_{\Omega}=\biggl\{ f\Big|f:\Omega\rightarrow \mathrm{Cl}_{0,n}(C), f(z)=\sum_{A}{f_{A}(z)e_{A}}, f_{A}(z)\in C^{r}(\Omega)\biggr\} }. $$

Dirac operators are introduced as follows [6]:

$$\begin{gathered} D^{l} f=\sum_{i=0}^{n}e_{i} \frac{\partial f}{\partial z_{i}};\qquad \overline{D^{l}}f=e_{0}{ \frac{\partial f }{\partial z_{0}}}-\sum_{i=1}^{n}e_{i}{ \frac{\partial f}{\partial z_{i}}}; \\ D^{r} f=\sum_{i=0}^{n} \frac{\partial f}{\partial z_{i}}e_{i};\qquad \overline{D^{r}}f={ \frac{\partial f }{\partial z_{0}}}e_{0}-\sum_{i=1}^{n}{ \frac{\partial f}{\partial z_{i}}}e_{i}. \end{gathered} $$

### Definition 2.1

([16])

If $\Omega\subset C^{n+1}$, $f: \Omega\rightarrow \mathrm{Cl}_{0,n}(C)$ satisfies:

1. $f_{A}(z)$ is a holomorphic function for any $z_{j}\in\Omega$,
2. $D_{l}f(z)=0$, $\forall z\in\Omega$,

then $f(z)$ is called a complex left regular function on Ω.

### Definition 2.2

([16])

If $\Omega\subset C^{n+1}$, $f: \Omega\rightarrow \mathrm{Cl}_{0,n}(C)$ satisfies:

1. $f_{A}(z)$ is a holomorphic function for any $z_{j}\in\Omega$,
2. $D_{r}f(z)=0$, $\forall z\in\Omega$,

then $f(z)$ is called a complex right regular function on Ω.

### Lemma 2.1

(Hadamard lemma [22])

*Let* $\Omega\subset R^{n+1}$ *be a bounded domain*, $n\geq2$. *If* *α*, *β* *satisfy* $0<\alpha,\beta <n+1$, *and* $\alpha+\beta>n+1$, *then for any* $x_{1},x_{2}\in R^{n+1}$, $x_{1}\neq x_{2}$, *we have*

$$\int_{\Omega} \vert t-x_{1} \vert ^{-\alpha} \vert t-x_{2} \vert ^{-\beta}\,dt\leq J_{1} \vert x_{1}-x_{2} \vert ^{(n+1)-\alpha-\beta}, $$

*where* $J_{1}$ *is a positive constant related to* *α* *and* *β*.

### Lemma 2.2

([22])

*Let* $\Omega\subset R^{n+1}$ *be a bounded domain*, *when* $\alpha< n+1$, *for any* $y\in R^{n+1}$, *we have*

$$\int_{\Omega} \vert x-y \vert ^{-\alpha}\,dx\leq M, $$

*where* *M* *is a positive constant only related to* *α* *and the size of* Ω.

### Lemma 2.3

(Hölder inequality [23])

*If* $f_{k}\in L^{p_{k}}(\Omega)$, $k=1,2,\ldots,n$, *and*

$${\frac{1}{p}=\frac{1}{p_{1}}+\frac{1}{p_{2}}+\cdots+\frac{1}{p_{n}} \leq1,} $$

*then* ${f_{1}f_{2}\cdots f_{n}\in L^{p}(\Omega)}$, *and*

$$L^{p}(f_{1}f_{2}\cdots f_{n})\leq L^{p_{1}}(f_{1})L^{p_{2}}(f_{2}) \cdots L^{p_{n}}(f_{n}), \quad p\geq1. $$

### Lemma 2.4

(Minkowski inequality [23])

*If* ${f_{1},f_{2},\ldots,f_{n}\in L^{p}(\Omega)}$, *then* $f_{1}+f_{2}+\cdots+f_{n}\in L^{p}(\Omega)$, *and*

$${L^{p}(f_{1}+f_{2}+\cdots+f_{n})\leq L^{p}(f_{1})+L^{p}(f_{2})+ \cdots+L^{p}(f_{n}), p\geq1.} $$

### Lemma 2.5

([23])

*Let* $L^{p}(\Omega,\mathrm{Cl}_{0,n}(R))$ *represent the set of all* *p* *order integrable functions which are defined on the bounded domain* $\Omega \subset R^{n+1}$, *and with values in the real Clifford algebra* $\mathrm{Cl}_{0,n}(R)$, *define the norm of* *φ* *as follows*:

$$\Vert \varphi \Vert _{\Omega,p}= \biggl( \int_{\Omega} \bigl\vert \varphi (x) \bigr\vert ^{p}\,dV_{x} \biggr)^{\frac{1}{p}},\quad p\geq1, $$

*when* $1\leq r \leq p$,

$$L^{p}\bigl(\Omega, \mathrm{Cl}_{0,n}(R)\bigr)\subset L^{r} \bigl(\Omega, \mathrm{Cl}_{0,n}(R)\bigr) $$

*is true*.

The notations used in this paper are as follows:

1. $\omega_{2n+2}$ represents the surface area of unit sphere in a $2n+2$-dimensional real Euclidean space.
2. $M_{i}$ $\{i=1,2,3\}$, $K_{i}$ $\{i=1,\dots,16\}$ are constants only related to *n* and the size of domain Ω in this paper.
3. $dV_{\xi}=d\zeta_{0}\wedge d\zeta_{1}\wedge\cdots\wedge d\zeta_{n}\wedge d\eta_{0}\wedge d\eta_{1}\wedge\cdots\wedge d\eta _{n}$, $\zeta_{j}\in R$, $\eta_{j}\in R$, ($j=0, 1, \ldots, n$), $\xi_{j}=\zeta _{j}+i\eta_{j}$.
4. $d\bar{\xi}\wedge d{\xi}=d\bar{\xi}_{0}\wedge d\bar{\xi}_{1}\wedge \cdots d\bar{\xi}_{n}\wedge d{\xi_{0}}\wedge d{\xi_{1}}\dots\wedge d{\xi_{n}}$.
5. $d\bar{\xi}\wedge d{\xi}=(2i)^{n+1}\,dV_{\xi}$.

## Some properties of a T operator with B-M kernel in the complex Clifford analysis

In this section, we discuss some properties of a singular integral operator.

### Definition 3.1

Let $\Omega\subset C^{n+1}$ be a bounded domain, $\varphi\in L^{p}(\overline{\Omega},\mathrm{Cl}_{0,n}(C))$, $z\in C^{n+1}$, then

$$\begin{aligned} &(T\varphi) (z) \\ &\quad=\frac{1}{\omega_{2n+2}(2i)^{n+1}} \int_{\Omega}\varphi(\xi) \biggl(\frac{\sum_{k=0}^{n}(\xi_{k}-z_{k})\bar{e}_{k}}{ \vert \xi-z \vert ^{2n+2}}+ \frac{\sum_{k=0}^{n}(\overline{\xi_{k}-z_{k}})\bar {e}_{k}}{ \vert \xi-z \vert ^{2n+2}} \biggr)\,d\bar{\xi}\wedge d{\xi} \end{aligned}$$

is called T operator with B-M kernel.

### Theorem 3.1

*Let* $\Omega\subset C^{n+1}$ *be a bounded domain*, $\varphi\in L^{p}(\overline{\Omega},\mathrm{Cl}_{0,n}(C))$, $p>n+1$, *then* *T* *is bounded on* $L^{p}(\Omega)$, *and*

1 $$\begin{aligned} \Vert T\varphi \Vert _{\Omega,p}\leq M_{1} \Vert \varphi \Vert _{\Omega,p}. \end{aligned}$$

### Proof

Choose $q>1$ such that $\frac{1}{p}+\frac{1}{q}=1$, when $p>2(n+1)$, we have $1< q<\frac{2(n+1)}{2n+1}$, using Hölder’s inequality, we have

$$\begin{aligned} & \bigl\vert T\varphi(z) \bigr\vert \\ &\quad= \frac{1}{2^{n+1}\omega_{2n+2}} \biggl\vert \int_{\Omega}\varphi(\xi ) \biggl(\frac{\sum_{k=0}^{n}(\xi_{k}-z_{k})\bar{e}_{k}}{ \vert \xi-z \vert ^{2n+2}}+ \frac{\sum_{k=0}^{n}(\overline{\xi_{k}-z_{k}})\bar {e}_{k}}{ \vert \xi-z \vert ^{2n+2}} \biggr)\,d\bar{\xi}\wedge d{\xi} \biggr\vert \\ &\quad\leq\frac{K_{1}}{\omega_{2n+2}} \biggl( \int_{\Omega} \biggl\vert \varphi (\xi)\frac{\sum_{k=0}^{n}(\xi_{k}-z_{k})\bar{e}_{k}}{ \vert \xi -z \vert ^{2n+2}} \biggr\vert \,dV_{\xi} + \int_{\Omega} \biggl\vert \varphi(\xi)\frac{\sum_{k=0}^{n}(\overline{\xi _{k}-z_{k}})\bar{e}_{k}}{ \vert \xi-z \vert ^{2n+2}} \biggr\vert \,dV_{\xi} \biggr) \\ &\quad\leq K_{2} \biggl( \int_{\Omega} \bigl\vert \varphi(\xi) \bigr\vert \frac{1}{ \vert \xi -z \vert ^{2n+1}}\,dV_{\xi}+ \int_{\Omega} \bigl\vert \varphi(\xi) \bigr\vert \frac{1}{ \vert \xi-z \vert ^{2n+1}} \,dV_{\xi} \biggr) \\ &\quad \leq K_{3} \int_{\Omega} \bigl\vert \varphi(\xi) \bigr\vert \frac{1}{ \vert \xi-z \vert ^{2n+1}}\,dV_{\xi} \\ &\quad\leq K_{4} \Vert \varphi \Vert _{\Omega,p} \biggl( \int_{\Omega} \vert \xi -z \vert ^{-(2n+1)q}\,dV_{\xi} \biggr)^{\frac{1}{q}}. \end{aligned}$$

Because $1< q<\frac{2(n+1)}{2n+1}$, we have $(2n+1)q<2(n+1)$. Using Lemma 2.2, for $\forall z\in\Omega$, we have

$$\int_{\Omega} \vert \xi-z \vert ^{-(2n+1)q}\,dV_{\xi} \leq K_{5}. $$

So we have

$$\bigl\vert T\varphi(z) \bigr\vert \leq K_{4}K_{5} \Vert \varphi \Vert _{\Omega,p}. $$

Hence,

$$\biggl( \int_{\Omega} \bigl\vert T\varphi(z) \bigr\vert ^{p}\,dV_{z} \biggr)^{\frac{1}{p}}\leq K_{4}K_{5} \biggl( \int_{\Omega} \Vert \varphi \Vert _{\Omega,p}^{p}\,dV_{z} \biggr)^{\frac{1}{p}}. $$

Let $M_{1}=K_{4}K_{5} (\int_{\Omega}\,dV_{z} )^{\frac{1}{p}}$, we have

$$\bigl\Vert T\varphi(z) \bigr\Vert _{\Omega,p}\leq M_{1} \Vert \varphi \Vert _{\Omega,p}. $$

 □

### Theorem 3.2

*Let* $z=z_{0}e_{0}+z_{1}e_{1}+z_{2}e_{2}+\cdots+z_{n}e_{n}$, $\xi=\xi _{0}e_{0}+\xi_{1}e_{1}+\xi_{2}e_{2}+\cdots+\xi_{n}e_{n}\in\mathrm{Cl}_{0,n}(C)$, $z\neq0$, $\xi\neq0$, *and* $|z|\neq|\xi|$, *n* (≥2), *m* (≥0) *be integers*, *then for any* *i*, $0\leq i\leq n$, *we have*

2 $$\begin{aligned} \biggl\vert \frac{z_{i}}{ \vert z \vert ^{m+2}}-\frac{\xi_{i}}{ \vert \xi \vert ^{m+2}} \biggr\vert \leq \frac{ \vert z-\xi \vert [P_{m}(z,\xi)+ \vert z \vert ^{\frac{m}{2}} \vert \xi \vert ^{\frac{m}{2}} ]}{ \vert z \vert ^{m+1} \vert \xi \vert ^{m+1}}, \end{aligned}$$

*where*

$$P_{m}(z,\xi)= \sum_{k=0}^{m} \vert z \vert ^{m-k} \vert \xi \vert ^{k}={ \frac{ \vert z \vert ^{m+1}- \vert \xi \vert ^{m+1}}{ \vert z \vert - \vert \xi \vert }}. $$

### Proof

Suppose $|z|\leq|\xi|$ and insert the term $z_{i}|z|^{m+2}$ in the following formula, then we have

$$\begin{aligned} & \biggl\vert \frac{z_{i}}{ \vert z \vert ^{m+2}}-\frac{\xi_{i}}{ \vert \xi \vert ^{m+2}} \biggr\vert \\ &\quad= \biggl\vert \frac{z_{i} \vert \xi \vert ^{m+2}-\xi_{i} \vert z \vert ^{m+2}}{ \vert z \vert ^{m+2} \vert \xi \vert ^{m+2}} \biggr\vert \\ &\quad= \biggl\vert \frac{z_{i} \vert \xi \vert ^{m+2}-z_{i} \vert z \vert ^{m+2}+z_{i} \vert z \vert ^{m+2}-\xi _{i} \vert z \vert ^{m+2}}{ \vert z \vert ^{m+2} \vert \xi \vert ^{m+2}} \biggr\vert \\ &\quad\leq\frac{ |z_{i}|| |\xi|^{m+2}- |z|^{m+2}|+|z_{i}-\xi _{i}| |z|^{m+2}}{ |z|^{m+2} |\xi|^{m+2}} \\ &\quad\leq\frac{|z|||\xi|-|z||(|\xi|^{m+1}+|\xi |^{m}|z|+\cdots+|z|^{m+1})+|z-\xi||z||\xi||z|^{m}}{|z|^{m+2}|\xi |^{m+2}} \\ &\quad\leq \biggl\vert \frac{|z|||\xi|-|z||(|\xi|^{m+1}+|\xi |^{m}|z|+\cdots+|\xi||z|^{m})+|z-\xi||z||\xi||z|^{m}}{|z|^{m+2}|\xi |^{m+2}} \biggr\vert \\ &\quad\leq \biggl\vert \frac{|z-\xi| [(|\xi|^{m}+|\xi|^{m-1}|z|+\cdots +|z|^{m})+|z|^{m} ]}{|z|^{m+1}|\xi|^{m+1}} \biggr\vert \\ &\quad\leq \biggl\vert \frac{|z-\xi| [P_{m}(z,\xi)+|z|^{\frac{m}{2}}|\xi |^{\frac{m}{2}} ]}{|z|^{m+1}|\xi|^{m+1}} \biggr\vert . \end{aligned}$$

When $|\xi|\leq|z|$, insert $\xi_{i}|\xi|^{m+2}$ in the above formula, we can prove the above inequality in a similar way. □

### Remark 1

Because the original Hile lemma cannot be used directly in the complex Clifford analysis, we give the conclusion of Theorem 3.2 which is similar to the classical Hile lemma and plays an important role in proving the properties of T-operators and Cauchy operators. We insert the appropriate items according to the situation and prove that inequality (2) holds. Inequality (2) is similar to the Hile lemma of the classical real vector and is complete symmetry with respect to the variable *ξ*, *z*. It is a good tool to prove the Hölder continuity of the T operator with B-M kernel in the complex Clifford analysis.

### Theorem 3.3

*Let* $\Omega\subset C^{n+1}$ *be a bounded domain*, $\varphi\in L^{p}(\Omega)$, $p>2(n+1)$, *then for any* $z_{1},z_{2}\in\Omega$, *we have*

3 $$\begin{aligned} \bigl\vert (T\varphi) (z_{1})-(T\varphi) (z_{2}) \bigr\vert \leq M_{2} \Vert \varphi \Vert _{\Omega,p} \vert z_{1}-z_{2} \vert ^{\alpha}, \end{aligned}$$

*and* *Tφ* *is Hölder continuous on* Ω, *where* $\alpha= 1-\frac{2(n+1)}{p}$.

### Proof

Case 1. When $|z_{1}-z_{2}|\geq1$, using Theorem 3.2 we have

$$\begin{aligned} \bigl\vert T\varphi(z_{1})-T\varphi(z_{2}) \bigr\vert \leq& 2M_{1} \Vert \varphi \Vert _{\Omega,p} \\ \leq& 2M_{1} \Vert \varphi \Vert _{\Omega,p} \frac {1}{ \vert z_{1}-z_{2} \vert ^{\alpha}} \vert z_{1}-z_{2} \vert ^{\alpha} \\ \leq& M_{2} \Vert \varphi \Vert _{\Omega,p} \vert z_{1}-z_{2} \vert ^{\alpha}. \end{aligned}$$

Case 2. When $|z_{1}-z_{2}|<1$, we have

$$\begin{aligned} & \bigl\vert T\varphi(z_{1})-T\varphi(z_{2}) \bigr\vert \\ &\quad\leq\frac{1}{\omega_{2n+2}2^{n+1}} \int_{\Omega} \bigl\vert \varphi(\xi ) \bigr\vert \biggl\vert \frac{\sum_{k=0}^{n}(\xi_{k}-z_{1k})\bar{e}_{k}}{ \vert \xi -z_{1} \vert ^{2n+2}} -\frac{\sum_{k=0}^{n}({\xi_{k}-z_{2k}})\bar{e}_{k}}{ \vert \xi -z_{2} \vert ^{2n+2}} \biggr\vert \vert d\bar{\xi}\wedge d{ \xi} \vert \\ &\qquad{}+ \frac{1}{\omega_{2n+2}2^{n+1}} \int_{\Omega} \bigl\vert \varphi(\xi ) \bigr\vert \biggl\vert \frac{\sum_{k=0}^{n}(\overline{\xi_{k}-z_{1k}})\bar{e}_{k}}{ \vert \xi -z_{1} \vert ^{2n+2}} -\frac{\sum_{k=0}^{n}(\overline{\xi_{k}-z_{2k}})\bar {e}_{k}}{ \vert \xi-z_{2} \vert ^{2n+2}} \biggr\vert \vert d\bar{\xi}\wedge d{ \xi} \vert \\ &\quad\leq\frac{1}{\omega_{2n+2}}\sum_{k=0}^{n} \int_{\Omega} \bigl\vert \varphi (\xi) \bigr\vert \biggl\vert \frac{(\xi_{k}-z_{1k})}{ \vert \xi-z_{1} \vert ^{2n+2}} -\frac{({\xi _{k}-z_{2k}})}{ \vert \xi-z_{2} \vert ^{2n+2}} \biggr\vert \,dV_{\xi} \\ &\qquad{}+\frac{1}{\omega_{2n+2}}\sum_{k=0}^{n} \int_{\Omega} \bigl\vert \varphi (\xi) \bigr\vert \biggl\vert \frac{(\overline{\xi_{k}-z_{1k}})}{ \vert \xi-z_{1} \vert ^{2n+2}} -\frac{(\overline{\xi_{k}-z_{2k}})}{ \vert \xi-z_{2} \vert ^{2n+2}} \biggr\vert \,dV_{\xi} \\ &\qquad=\frac{2}{\omega_{2n+2}}\sum_{k=0}^{n} \int_{\Omega} \bigl\vert \varphi(\xi ) \bigr\vert \biggl\vert \frac{(\xi_{k}-z_{1k})}{ \vert \xi-z_{1} \vert ^{2n+2}} -\frac{({\xi _{k}-z_{2k}})}{ \vert \xi-z_{2} \vert ^{2n+2}} \biggr\vert \,dV_{\xi}. \end{aligned}$$

Let

$$I= \int_{\Omega} \bigl\vert \varphi(\xi) \bigr\vert \biggl\vert \frac{(\xi_{k}-z_{1k})}{ \vert \xi -z_{1} \vert ^{2n+2}} -\frac{({\xi_{k}-z_{2k}})}{ \vert \xi-z_{2} \vert ^{2n+2}} \biggr\vert \,dV_{\xi}. $$

According to Theorem 3.2, we can get

$$\begin{aligned} I \leq& \int_{\Omega} \bigl\vert \varphi(\xi) \bigr\vert \biggl\vert \frac{ \vert z_{1}-z_{2} \vert [P_{2n}(\xi-z_{1},\xi-z_{2})+ \vert \xi-z_{1} \vert ^{n} \vert \xi-z_{2} \vert ^{n} ]}{ \vert \xi -z_{1} \vert ^{2n+1} \vert \xi-z_{2} \vert ^{2n+1}} \biggr\vert \,dV_{\xi} \\ =& \vert z_{1}-z_{2} \vert \int_{\Omega} \bigl\vert \varphi(\xi) \bigr\vert \biggl\vert \frac{P_{2n}(\xi -z_{1},\xi-z_{2})}{ \vert \xi-z_{1} \vert ^{2n+1} \vert \xi-z_{2} \vert ^{2n+1}} \biggr\vert \,dV_{\xi } \\ &{}+ \vert z_{1}-z_{2} \vert \int_{\Omega} \bigl\vert \varphi(\xi) \bigr\vert \frac{ \vert \xi-z_{1} \vert ^{n} \vert \xi -z_{2} \vert ^{n}}{ \vert \xi-z_{1} \vert ^{2n+1} \vert \xi-z_{2} \vert ^{2n+1}}\,dV_{\xi} \\ =&I_{1}+I_{2}. \end{aligned}$$

For $I_{1}$, we have

$$\begin{aligned} I_{1} =& \vert z_{1}-z_{2} \vert \int_{\Omega}\sum_{k=0}^{2n} \vert \xi-z_{1} \vert ^{-(k+1)}|\xi-z_{2})|^{-(2n+1-k)}| \varphi(\xi)|dV_{\xi} \\ =& \vert z_{1}-z_{2} \vert \sum _{k=0}^{2n} \int_{\Omega} \vert \xi-z_{1} \vert ^{-(k+1)}| \xi-z_{2})|^{-(2n+1-k)}|\varphi(\xi)|dV_{\xi}. \end{aligned}$$

Using Hölder’s inequality we have

$$I_{1}\leq \vert z_{1}-z_{2} \vert \Vert \varphi \Vert _{\Omega,p} \sum_{k=0}^{2n} \biggl( \int_{\Omega} \vert \xi-z_{1} \vert ^{-(k+1)q} \vert \xi -z_{2} \vert ^{-(2n+1-k)q} \,dV_{\xi} \biggr)^{\frac{1}{q}}. $$

Because $p>2n+2$, $\frac{1}{p}+\frac{1}{q}=1$, we can get

$$1< q< \frac{2n+2}{2n+1}. $$

So

$$2n+1< (2n+1)q< 2n+2, $$

$0\leq k\leq2n$, we get

$$\begin{gathered} (k+1)q\leq2n+2, \\ (2n+1-k)q\leq2n+2, \end{gathered} $$

and

$$(k+1)q+(2n+1-k)q>2n+2. $$

By Hadamard’s lemma, we have

$$\begin{aligned} & \int_{\Omega} \vert \xi-z_{1} \vert ^{-(k+1)q}| \xi-z_{2})|^{-(2n+1-k)q}\,dV_{\xi} \\ &\quad\leq K_{6} \vert z_{1}-z_{2} \vert ^{(2n+2)-(2n+1-k)q-(k+1)q} \\ &\quad=K_{6} \vert z_{1}-z_{2} \vert ^{(2n+2)-(2n+2)q}. \end{aligned}$$

So we have

$$\begin{aligned} I_{1} \leq& (2n+1)K_{6} \Vert \varphi \Vert _{\Omega,p} \vert z_{1}-z_{2} \vert ^{1+\frac{2n+2}{q}-(2n+2)} \\ \leq&(2n+1)K_{6} \Vert \varphi \Vert _{\Omega,p} \vert z_{1}-z_{2} \vert ^{1-\frac{2n+2}{p}}. \end{aligned}$$

As to $I_{2}$, using Hölder’s inequality we have

$$\begin{aligned} I_{2} =& \vert z_{1}-z_{2} \vert \int_{\Omega} \bigl\vert \varphi(\xi) \bigr\vert \frac{ \vert \xi -z_{1} \vert ^{n} \vert \xi-z_{2} \vert ^{n}}{ \vert \xi-z_{1} \vert ^{2n+1} \vert \xi-z_{2} \vert ^{2n+1}}\,dV_{\xi } \\ \leq& \vert z_{1}-z_{2} \vert \Vert \varphi \Vert _{\Omega,p} \biggl( \int_{\Omega } \vert \xi-z_{1} \vert ^{-(n+1)q} \vert \xi-z_{2} \vert ^{-(n+1)q} \,dV_{\xi} \biggr)^{\frac{1}{q}}. \end{aligned}$$

Since $p>2n+2$ and $\frac{1}{p}+\frac{1}{q}=1$, we get

$$1< q< \frac{2n+2}{2n+1}. $$

So

$$\begin{gathered} (n+1)q\leq2n+2, \\ (2n+2)q\geq2n+2. \end{gathered} $$

From Hadamard’s lemma, we get

$$\begin{aligned} & \int_{\Omega} \vert \xi-z_{1} \vert ^{-(n+1)q} \vert \xi-z_{2} \vert ^{-(n+1)q} \,dV_{\xi} \\ &\quad\leq K_{7} \vert z_{1}-z_{2} \vert ^{(2n+2)-(2n+2)q}. \end{aligned}$$

So

$$\begin{aligned} I_{2} \leq& K_{7} \Vert \varphi \Vert _{\Omega,p} \vert z_{1}-z_{2} \vert ^{1+\frac{2n+2}{q}-(2n+2)} \\ \leq& K_{7} \Vert \varphi \Vert _{\Omega,p} \vert z_{1}-z_{2} \vert ^{1-\frac{2n+2}{p}}. \end{aligned}$$

Hence

$$\begin{aligned} I =&I_{1}+I_{2} \\ \leq& (2n+1) (K_{6}+K_{7}) \Vert \varphi \Vert _{\Omega,p} \vert z_{1}-z_{2} \vert ^{1-\frac{2n+2}{p}} \\ =&K_{8} \Vert \varphi \Vert _{\Omega,p} \vert z_{1}-z_{2} \vert ^{1-\frac {2n+2}{p}}. \end{aligned}$$

Using Hölder’s inequality, we obtain

$$\begin{aligned} \bigl\vert T\varphi(z_{1})-T\varphi(z_{2}) \bigr\vert \leq&\frac{2}{\omega_{2n+2}} K_{8} \Vert \varphi \Vert _{\Omega,p} \vert z_{1}-z_{2} \vert ^{1-\frac{2n+2}{p}} \\ \leq&K_{9} \Vert \varphi \Vert _{\Omega,p} \vert z_{1}-z_{2} \vert ^{1-\frac{2n+2}{p}} \\ \leq&M_{2} \Vert \varphi \Vert _{\Omega,p} \vert z_{1}-z_{2} \vert ^{\alpha}. \end{aligned}$$

 □

### Remark 2

In Case 2 of this theorem, we use the inequality of Theorem 3.3, Hölder’s inequality, and Hadamard’s lemma. This result enriches the theoretical system of the complex Clifford analysis.

### Theorem 3.4

*Let* $\Omega\subset C^{n+1}$ *be a bounded domain*, $\varphi\in L^{p}(\Omega)$, $1< p<2n+2$, *γ* *is an arbitrary constant which satisfies* $1<\gamma<\frac{(2n+2)p}{(2n+2)-p}$, *then* *Tφ* *is* *γ*-*integrable on* Ω, *that is*, $T\varphi \in L^{\gamma}(\Omega)$, *and the following inequality*

4 $$\begin{aligned} \Vert T\varphi \Vert _{\Omega,\gamma}\leq M_{3} \Vert \varphi \Vert _{\Omega,p} \end{aligned}$$

*is true*.

### Proof

For convenience, we introduce the notation *b*, suppose $b=\frac {1}{\gamma}-\frac{1}{p}+\frac{1}{2n+2}$, then from$1<\gamma<\frac{(2n+2)p}{(2n+2)-p}$ we know $b>0$. Here are two cases to prove that *Tφ* is *γ*-integrable on Ω.

Case 1. When $p<\gamma<\frac{(2n+2)p}{(2n+2)-p}$, $0<\frac{p}{\gamma }<1$, thus $0< p(\frac{1}{p}-\frac{1}{\gamma})=1-\frac{p}{\gamma}<1$, again

$$\frac{p}{\gamma}+p\biggl(\frac{1}{p}-\frac{1}{\gamma}\biggr)=1. $$

Choose $q>0$ such that $\frac{1}{p}+\frac{1}{q}=1$, then we have

$$\begin{aligned} &(2n+2) \biggl(\frac{b}{2}-\frac{1}{\gamma}\biggr)+(2n+2) \biggl( \frac{b}{2}-\frac{1}{q}\biggr) \\ &\quad=(2n+2) \biggl(b-\frac{1}{\gamma}-\frac{1}{q}\biggr) \\ &\quad=(2n+2) \biggl(\frac{1}{\gamma}-\frac{1}{p}+\frac{1}{2n+2}- \frac {1}{\gamma}-\frac{1}{q}\biggr) \\ &\quad=(2n+2) \biggl(-1+\frac{1}{2n+2}\biggr) \\ &\quad=-(2n+1). \end{aligned}$$

Therefore,

$$\begin{aligned} & \bigl\vert T\varphi(z) \bigr\vert \\ &\quad= \frac{1}{2^{n+1}\omega_{2n+2}} \biggl\vert \int_{\Omega}\varphi(\xi ) \biggl(\frac{\sum_{k=0}^{n}(\xi_{k}-z_{k})\bar{e}_{k}}{ \vert \xi-z \vert ^{2n+2}}+ \frac{\sum_{k=0}^{n}(\overline{\xi_{k}-z_{k}})\bar{e}_{k}}{ \vert \xi -z \vert ^{2n+2}} \biggr)\,d\bar{\xi}\wedge d{\xi} \biggr\vert \\ &\quad\leq\frac{K_{10}}{\omega_{2n+2}} \biggl( \int_{\Omega} \bigl\vert \varphi (\xi) \bigr\vert \frac{1}{ \vert \xi-z \vert ^{2n+1}}\,dV_{\xi} + \int_{\Omega} \bigl\vert \varphi (\xi) \bigr\vert \frac{1}{ \vert \xi-z \vert ^{2n+1}}\,dV_{\xi} \biggr) \\ &\quad= \frac{2K_{10}}{\omega_{2n+2}} \int_{\Omega} \bigl\vert \varphi(\xi ) \bigr\vert \frac{1}{ \vert \xi-z \vert ^{2n+1}}\,dV_{\xi} \\ &\quad= \frac{2K_{10}}{\omega_{2n+2}} \int_{\Omega} \bigl\vert \varphi(\xi ) \bigr\vert ^{\frac{p}{\gamma}} \vert \xi-z \vert ^{(2n+2)(\frac{b}{2}-\frac{1}{\gamma})} \bigl\vert \varphi(\xi) \bigr\vert ^{p(\frac{1}{p}-\frac{1}{\gamma})} \vert \xi -z \vert ^{(2n+2)(\frac{b}{2}-\frac{1}{q})}\,dV_{\xi}. \end{aligned}$$

Because $1< p<\gamma$, $\frac{1}{\gamma}+(\frac{1}{p}-\frac{1}{\gamma })+\frac{1}{q}=1$, using Hölder’s inequality we get

$$\begin{aligned} & \bigl\vert T\varphi(z) \bigr\vert \\ &\quad\leq\frac{2K_{10}}{\omega_{2n+2}} \biggl( \int_{\Omega} \bigl\vert \varphi(\xi) \bigr\vert ^{p} \vert \xi-z \vert ^{(2n+2)(\frac{\gamma b}{2}-1)}\,dV_{\xi } \biggr)^{\frac{1}{\gamma}} \biggl( \int_{\Omega} \bigl\vert \varphi(\xi) \bigr\vert ^{p}\,dV_{\xi} \biggr)^{\frac {1}{p}-\frac{1}{\gamma}} \\ & \qquad{} \cdot \biggl( \int_{\Omega} \vert \xi-z \vert ^{(2n+2)(\frac {qb}{2}-1)}\,dV_{\xi} \biggr)^{\frac{1}{q}} \\ &\quad= \frac{2K_{10}}{\omega_{2n+2}} \biggl( \int_{\Omega} \bigl\vert \varphi (\xi) \bigr\vert ^{p} \vert \xi-z \vert ^{(2n+2)(\frac{\gamma b}{2}-1)}\,dV_{\xi} \biggr)^{\frac{1}{\gamma}} \Vert \varphi \Vert _{\Omega,p}^{1-\frac{p}{\gamma }} \\ & \qquad{} \cdot \biggl( \int_{\Omega} \vert \xi-z \vert ^{(2n+2)(\frac {qb}{2}-1)}\,dV_{\xi} \biggr)^{\frac{1}{q}}. \end{aligned}$$

Because $b>0$, we have

$$\begin{gathered} (2n+2) \biggl(1-\frac{\gamma b}{2}\biggr)< 2n+2, \\ (2n+2) \biggl(1-\frac{qb}{2}\biggr)< 2n+2. \end{gathered} $$

From Lemma 2.2 we can know that two integrals are meaningful, we assume that $K_{11}= \sup_{\xi\in\Omega} \int_{\Omega}|\xi -z|^{(2n+2)(\frac{qb}{2}-1)}\,dV_{\xi}$.

Therefore we have

$$\bigl\vert T\varphi(z) \bigr\vert ^{\gamma}\leq \biggl( \frac{2}{\omega_{2n+2}} \biggr)^{\gamma }K_{11}^{\frac{\gamma}{q}} \Vert \varphi \Vert _{\Omega,p}^{\gamma-p} \biggl( \int_{\Omega} \bigl\vert \varphi(\xi) \bigr\vert ^{p} \vert \xi-z \vert ^{(2n+2)(\frac{\gamma b}{2}-1)}\,dV_{\xi } \biggr). $$

Let $K_{12}= \sup_{\xi\in\Omega} \int_{\Omega}|\xi -z|^{(2n+2)(\frac{\gamma b}{2}-1)}\,dV_{z}$, so we have

$$\bigl\vert T\varphi(z) \bigr\vert ^{\gamma}\leq K_{12} \Vert \varphi \Vert _{\Omega,p}^{\gamma -p} \Vert \varphi \Vert _{\Omega,p}^{p}=K_{13} \Vert \varphi \Vert _{\Omega,p}^{\gamma}, $$

where $K_{13}= (\frac{2}{\omega_{2n+2}} )^{\gamma}K_{11}^{\frac {\gamma}{q}}K_{12}$.

Hence, we get

$$\Vert T\varphi \Vert _{\Omega,\gamma}= \biggl( \int_{\Omega} \bigl\vert T\varphi (z) \bigr\vert ^{\gamma}\,dV_{\xi} \biggr)^{\frac{1}{\gamma}} \leq K_{13}^{\gamma} \Vert \varphi \Vert _{\Omega,p}=K_{14} \Vert \varphi \Vert _{\Omega,p}, $$

where $K_{14}= K_{13}^{\gamma}$.

(2) When $p\geq\gamma>1$, choose *m* such that $0<\frac{(2n+2)\gamma }{(2n+2)+\gamma}<m<\gamma$, and *m* is an arbitrary positive constant satisfying $m<\gamma<\frac {(2n+2)m}{(2n+2)+m}$. Because $\varphi\in L^{p}(\Omega)$, $m< p$, we have $\varphi\in L^{m}(\Omega)$.

Choose $\frac{1}{p}+\frac{1}{q}=\frac{1}{m}$. Therefore, from the proof process of (1) and Lemma 2.5, we get

$$\begin{aligned} & \Vert T\varphi \Vert _{\Omega,p} \\ &\quad\leq K_{15} \Vert \varphi \Vert _{\Omega,m} \\ &\quad= K_{15} \biggl[ \int_{\Omega} \bigl\vert \varphi(\xi)\cdot1 \bigr\vert ^{m} \biggr]^{\frac{1}{m}} \\ &\quad\leq K_{15} \biggl[ \int_{\Omega} \bigl\vert \varphi(\xi)\cdot1 \bigr\vert ^{p} \biggr]^{\frac{1}{p}} \biggl[ \int_{\Omega} \bigl\vert \varphi(\xi)\cdot1 \bigr\vert ^{q} \biggr]^{\frac{1}{q}} \\ &\quad\leq K_{15}V_{\Omega}^{\frac{1}{q}} \Vert \varphi \Vert _{\Omega,p} \\ &\quad\leq K_{16} \Vert \varphi \Vert _{\Omega,p}. \end{aligned}$$

Therefore *Tφ* is *γ* integrable on Ω. If we choose $M_{3}=\max\{K_{14},K_{16}\}$, then

$$\Vert T\varphi \Vert _{\Omega,\gamma}\leq M_{3} \Vert \varphi \Vert _{\Omega,p}. $$

 □
